# Observation of the low frequency vibrational modes of bacteriophage M13 in water by Raman spectroscopy

**DOI:** 10.1186/1743-422X-3-79

**Published:** 2006-09-22

**Authors:** KT Tsen, Eric C Dykeman, Otto F Sankey, Nien-Tsung Lin, Shaw-Wei D Tsen, Juliann G Kiang

**Affiliations:** 1 Department of Physics and Astronomy, Arizona State University, Tempe, AZ 85287-1504, USA; 2 Institute of Microbiology, Immunology and Molecular Medicine, Tzu Chi University, 701, Sec. 3, Chung-Yang Rd., Hualien 970, Taiwan, R.O.C; 3 Department of Pathology, Johns Hopkins Medical Institutions, Baltimore, MD 21231, USA; 4 Department of Cellular Injury, Walter Reed Army Institute of Research, Silver Spring, MD 20910-7500, USA; 5 Department of Medicine, Uniformed Services University of The Health Sciences, Bethesda, MD 20814-4799, USA; 6 Department of Pharmacology, Uniformed Services University of The Health Sciences, Bethesda, MD 20814-4799, USA

## Abstract

**Background:**

Recently, a technique which departs radically from conventional approaches has been proposed. This novel technique utilizes biological objects such as viruses as nano-templates for the fabrication of nanostructure elements. For example, rod-shaped viruses such as the M13 phage and tobacco mosaic virus have been successfully used as biological templates for the synthesis of semiconductor and metallic nanowires.

**Results and discussion:**

Low wave number (≤ 20 *cm*^-1^) acoustic vibrations of the M13 phage have been studied using Raman spectroscopy. The experimental results are compared with theoretical calculations based on an elastic continuum model and appropriate Raman selection rules derived from a bond polarizability model. The observed Raman mode has been shown to belong to one of the Raman-active axial torsion modes of the M13 phage protein coat.

**Conclusion:**

It is expected that the detection and characterization of this low frequency vibrational mode can be used for applications in nanotechnology such as for monitoring the process of virus functionalization and self-assembly. For example, the differences in Raman spectra can be used to monitor the coating of virus with some other materials and nano-assembly process, such as attaching a carbon nanotube or quantum dots.

## Background

Although viruses are generally regarded as etiologic agents of disease, they can also be made useful, and the concept of using them as a template to build uniform semiconductor nanostructures seems to be increasingly plausible. Among the crucial issues associated with molecular-beam epitaxy (MBE) self-assembly for applications in nanotechnology is the variation in component size [1,2]. The production of identical or nearly identical structures at nanoscale is desirable; however, this is extremely difficult to accomplish with conventional techniques. To date, MBE-grown nanowires, nanorods, and quantum dots, which are proposed to be elements in future nanoelectronic circuits, have been characterized by size dispersion despite numerous efforts towards the control of size and shape [3,4]. Although the method of self-assembly by employment of chemically generated templates [5,6] decreases to some extent the size dispersion of the grown structures, the results are still not satisfactory.

Recently, a technique which departs radically from conventional approaches has been proposed [7-11]. This novel technique utilizes biological objects such as viruses as nano-templates for the fabrication of nanostructure elements. For example, rod-shaped viruses such as the M13 phage and tobacco mosaic virus (TMV) have been successfully used as biological templates for the synthesis of semiconductor and metallic nanowires [7,10,11]. Furthermore, genetically modified TMV and M13 phage have been shown to be useful for the self-assembly of nanomaterials into liquid crystals, films and fibers [8,9]. It is therefore very likely that genetically programmed viruses will play an important role in developing the next generation of optoelectronic devices and nanoelectronic circuits.

For monitoring the abovementioned self-assembly processes, an *in-situ*, non-destructive technique is desirable. Raman spectroscopy has been shown to be a non-invasive technique in material research. To the authors' knowledge, previous studies of viruses using Raman spectroscopy have focused only on the high frequency (large wave number) regions (≥ 600 *cm*^-1^) where the internal virus composition, i.e. localized vibrations of multiply bonded or electron-rich groups in proteins, was studied [12]. In this paper, we report the first observation of low wave number (≤ 20 *cm*^-1^) acoustic vibrations of the M13 phage using Raman spectroscopy. The observed vibrations are compared with theoretical calculations based on an elastic continuum model and appropriate Raman intensities and selection rules derived from a continuum limit of the bond polarizability model. The observed Raman mode has been shown to belong to one of the Raman-active axial torsion modes of the M13 phage protein coat. Because of the sensitivity of these frequencies upon environments, it is expected that the detection of this low frequency vibrational mode can be used to monitor and help to control the process of virus functionalization, By virus functionalization we mean virus being used to make useful things such as nanostructure and nanoelectronic devices; for instance, when coating viruses with different materials, attaching viruses to quantum dots and carbon nanotubes, and forming multiple superstructures.

## Samples and experimental technique

The M13 phage samples in water solution used in this work were prepared as follows: To propagate the M13 phage, an overnight culture of the host cell, JM101, was diluted 20-fold into 125-ml flasks containing 20 ml of LB medium. When the culture reached 0.5 of optical density at 550 nm (OD_550_), the phage was added at a multiplicity of infection of 20 and further grown until stationary phase (ca. 12 h postinfection). Crude phage suspensions were prepared by centrifugation (10,000 × *g*, 15 min) of the culture to remove the cells and passing the supernatants through a membrane filter (0.45-μm pore size). To concentrate the phage titer, the filtrated supernatant was precipitated by 0.25 M NaCl and 2.5% polyethylene glycol 6000 for 4 hr on ice. The pellet collected by centrifugation (13500 × g for 15 min at 4°C) was dissolved in 100 μl of distilled water. To determine the phage titer, a double-layer bioassay [13] was performed on an LB agar plate.

To prevent heating of the samples by laser irradiation during the Raman scattering experiments, the second harmonic output of a cw mode-locked YAlG laser was used as an excitation source [14]. The laser, which has photon energy of 2.34 eV, was operated at a repetition rate of 76 MHz and has a pulse width of about 70 ps. 90°-scattering geometry was employed. The Raman scattered signal was collected and analyzed by a standard computer-controlled Raman system which included a double spectrometer and a photomultiplier tube with associated photon counting electronics. The spectrometer had a spectral resolution of about 1.0 *cm*^-1^. All the data reported here were taken at T = 300K.

## Experimental results, theoretical model calculations and discussions

**Figure 1:**
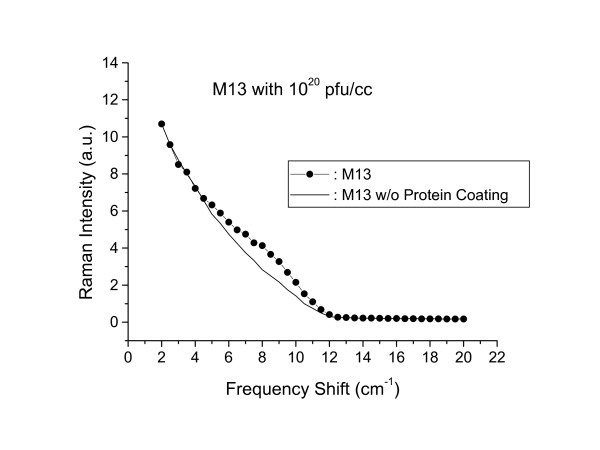
Raman scattering spectrum of M13 phages in water for a concentration of 10^20 ^pfu/ml with (solid circles) and without (solid curve) protein coating. The solid curve represents background signal resulting from the imperfection of rejection of elastic scattering of light by spectrometer.

**Figure 2:**
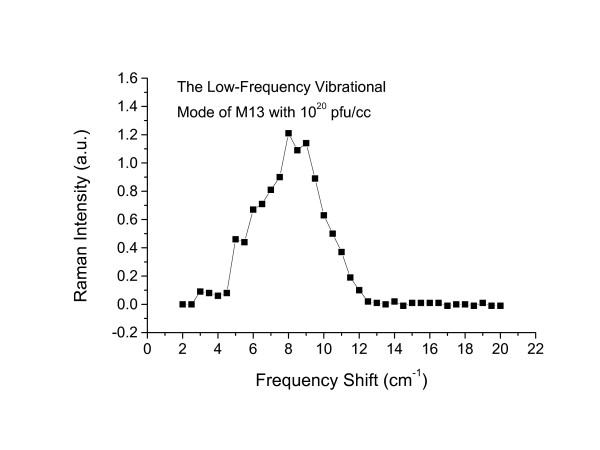
The low frequency vibrational mode of M13 with a concentration of 10^20 ^pfu/ml obtained after the subtraction of the background signal.

A typical Raman scattering spectrum taken for M13 phages at 10^20 ^pfu/ml and in the spectral range between 2 and 20 *cm*^-1 ^is shown in Fig. 1 (the solid circles). The experiments have been repeated at least 30 times and the data have been found to be reproducible. They were an average of at least 30 experiments. The statistic error (mostly from that of photon counting) associated with the data is minimal. The distinctive feature of the spectrum is a broad shoulder around 8.5 *cm*^-1 ^sitting on top of a background. To rule out both instrumental artifacts and the possibility of contributions from the DNA within the phages, we repeated the experiments with M13 phages without protein coats, i.e. with only the single-stranded M13 phage DNA at the same concentration in water. We notice that from the dimension of M13 phage and the weight of a single stranded DNA inside the phage, one can determine the equivalent weight of single stranded DNA for M13 phages with 10^20 ^and 10^21 ^pfu/ml. It is this equivalent weight of single stranded DNA that was used in the experiments. The results are shown as a solid line in Fig. 1. Comparison of the two spectra shows that there is indeed a broad structure at 8.5 *cm*^-1 ^associated with scattering of light from the M13 phage protein coats. The remaining background is due to imperfections in the rejection of elastic light by the spectrometer. The actual low frequency acoustic vibrational mode signal from the phages is obtained by the subtraction of these two spectra. Figure 2 shows the resulting structure after the subtraction. The broad peak has been found to center around 8.5 *cm*^-1 ^and is asymmetric. It has a full-width-at-half-maximum (FWHM) of about 5.0 *cm*^-1^. Since the spectral resolution of our Raman system is about 1.0 *cm*^-1^, we conclude that the relatively broad Raman peak observed here likely resulted from inhomogeneous broadening.

**Figure 3:**
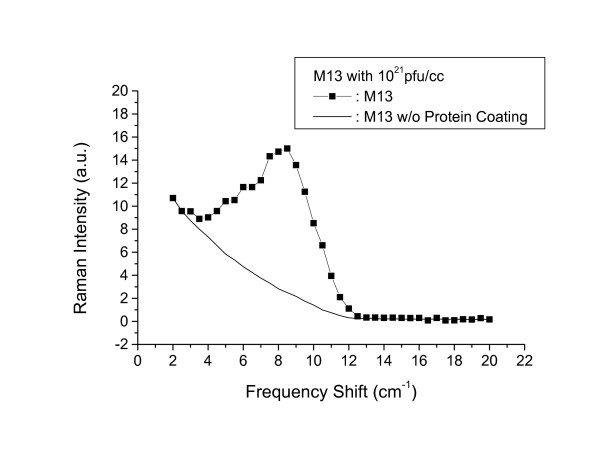
Raman scattering spectrum of M13 phages in water for a concentration of 10^21 ^pfu/ml with (solid circles) and without (solid curve) protein coating. The solid curve corresponds to background signal due to the imperfection of rejection of elastic scattering of light by spectrometer.

**Figure 4:**
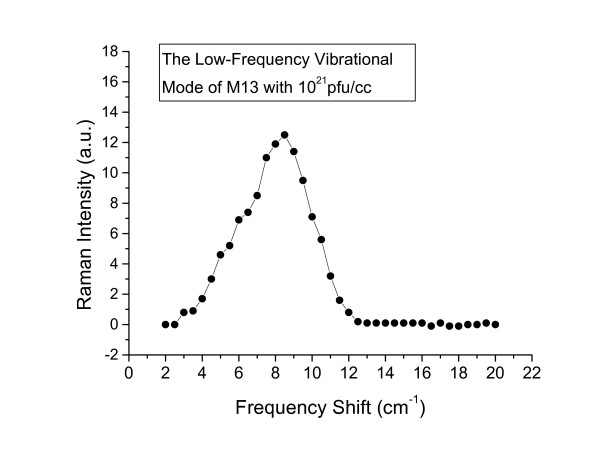
The low frequency vibrational mode of M13 with a concentration of 10^21 ^pfu/ml obtained after the subtraction of the background signal.

In order to further test our interpretations, we carried out similar Raman experiments with M13 phages at the higher concentration of 10^21 ^pfu/ml in water solution. The result is shown in Fig. 3. Again, the solid squares and solid line refer to the results for M13 phages with and without protein coats, respectively. Fig. 4 shows the resulting structure after the subtraction. Similar to Fig. 2, the observed broad Raman peak is centered around 8.5 *cm*^-1 ^and is asymmetric; however, the signal-to-noise ratio is significantly improved. This further confirms that what we have observed is the low frequency acoustic vibrational mode associated with the M13 phage protein coat.

**Figure 5:**
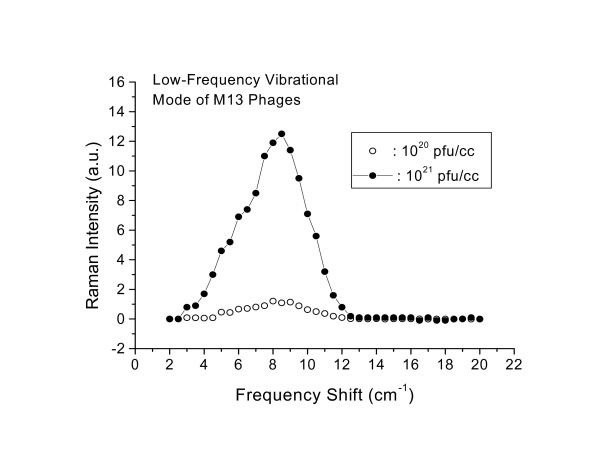
Raman spectra of low frequency vibrational mode of M13 phages for two concentrations, as indicated. The Raman intensity has been found to scale with theconcentration of the phages, as expected.

Fig. 5 shows a comparison of the observed broad structures for the two concentrations studied, 10^20 ^pfu/ml and 10^21 ^pfu/ml. The data are properly normalized. We find that the integrated area under the peaks scales very well with M13 phage concentration, as expected.

To obtain better insight into the character of the observed low frequency vibrational Raman mode of M13 phages, we performed theoretical calculations in which an elastic continuum model is assumed and appropriate Raman selection rules were derived based on a continuum extension of the bond polarizability model.

Experimental Raman spectroscopy is able to observe a low frequency mode of a cylindrical type viral particle; however, it is unable to provide a qualitative picture of the type of atomic displacements involved in the vibration. To understand what types of modes are being excited by the Raman spectroscopy experiment, we turn to a theoretical analysis of the problem.

There are several routes that can be followed in developing a theory capable of predicting Raman spectral lines, including atomistic models which give full atomic detail, to a continuum model which averages over the local geometry on the atomic scale and expands its vision to a larger length scale. Low frequency modes being probed here through Raman scattering have long wavelengths and thus are not sensitive to atomic details. Rather they are the result of the average global properties of the phage. Our goal here is to provide qualitative insights and a semi-quantitative understanding concerning the types of vibrational modes accessible through Raman scattering of a cylindrical phage. Thus a continuum approach, when used cautiously, is a method of choice due to its relative simplicity and ease of interpretation.

**Figure 6:**
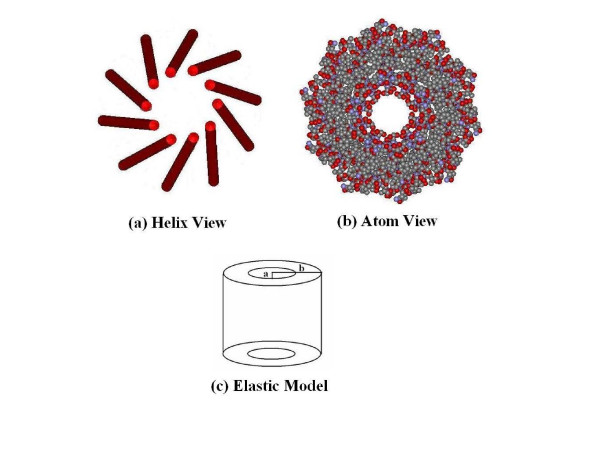
Diagrams of the structure of M13 phage. (a) A molecular model containing 10 α-helix coat proteins forming a cone of the structure. (b) A cross section of the phage composed of α-helix coat proteins. (c) The elastic continuum model of M13; a circular cylinder of outer radius "b" and inner radius "a."

In building a continuum model, it is important to first understand the structure of the M13 bacteriophage. M13 is composed of individual coat proteins bundled together so that in cross section the bundle has approximately the shape of a long circular cylinder (>860 nm), or tube [15]. Each individual coat protein in the bundle is comprised of 50 amino acids and has the conformation of an α-helix roughly aligned with the tube axis. Figure 6 shows a small segment of the filament. In cross section, there are 10 individual proteins packed together. The coat proteins are packed in a partially helical fashion so that they form the shape of a cone. The narrow neck at the top of one cone fits inside the large base of the cone above it. One end of each cone contains acidic residues while the other end is basic. This produces strong cone-cone interactions which increase the stability of the assembly. The central region of the cone is apolar and enhances the interactions between α-helices of a single cone. The stacking of thousands of such cones gives the phage its long worm-like structure. Inside the core of the tube resides the ssDNA and water.

We model the long fiber as a solid cylindrical tube of elastic continuous material, as shown in Figure 6c. Following Balandin et al. [16] the viral protein coat has an inner radius "a" and outer radius "b". The inner and outer radii of the M13 shell are set to a = 1.9 nm and b = 3.4 nm, respectively, which were determined from the atomistic structure of the capsid by averaging over distances between atoms in the structure. Elastic wave theory [17,18] is used to arrive at an analytic expression that describes the frequencies and displacement patterns of each vibrational mode of the cylindrical shell. Cylindrical coordinates (r, θ, z) are used where z is the direction along the axis of the cylinder. In general, the vibrational modes depend on the wave vector k_Z _varying as *e*^*ik*^*z*^*Z*^. We assume that the viral particle is longer than the wavelength of the light; Raman scattering is then only produced by waves with *k*_*z *_≈ 0. The displacement vectors of the vibrational modes are then u→
 MathType@MTEF@5@5@+=feaafiart1ev1aaatCvAUfKttLearuWrP9MDH5MBPbIqV92AaeXatLxBI9gBaebbnrfifHhDYfgasaacH8akY=wiFfYdH8Gipec8Eeeu0xXdbba9frFj0=OqFfea0dXdd9vqai=hGuQ8kuc9pgc9s8qqaq=dirpe0xb9q8qiLsFr0=vr0=vr0dc8meaabaqaciaacaGaaeqabaqabeGadaaakeaacuWG1bqDgaWcaaaa@2E31@(*r*, θ) with no z dependence (*e*^*ik*^*z*^*Z *^= 1), and with components u_r _(radial direction), u_θ _(angular direction) and u_Z _(axial direction). The radial dependence is a linear combination of Bessel functions of the 1^st ^and 2^nd ^kinds, and the angular dependence is sinusoidal being proportional to sin(nθ) and cos(nθ) where n is an integer, n = [0,∞]. Since the net radial force is zero on the surfaces of the shell, the radial stress components *σ*_*rr*_, *σ*_*rθ*_, and *σ*_*rz *_must vanish at the inner and outer radii "a" and "b." This gives six conditions relating the amplitudes that produce a 6 × 6 determinant^17 ^which must vanish at the allowed frequencies ω. There are just two parameters in the theory, and they are the longitudinal and transverse sound speeds, *c*_*l *_and *c*_*t*_. We use *c*_*l *_= 1817 m/s and *c*_*t *_= 915 m/s, the measured sound speeds in lysozymes [19], which is similar to the measured speeds of sound in different protein crystals such as ribonuclease and hemoglobin.

Elastic wave theory provides a complete description of the possible frequencies and their displacement patterns of vibrational modes which are then used to determine their Raman scattering intensity. The Raman intensity is proportional to the Raman tensor Δ_*χ *_as [20]

I∝|d^s·(Δχ↔)·d^i|2     Eq. 1,
 MathType@MTEF@5@5@+=feaafiart1ev1aaatCvAUfKttLearuWrP9MDH5MBPbIqV92AaeXatLxBI9gBaebbnrfifHhDYfgasaacH8akY=wiFfYdH8Gipec8Eeeu0xXdbba9frFj0=OqFfea0dXdd9vqai=hGuQ8kuc9pgc9s8qqaq=dirpe0xb9q8qiLsFr0=vr0=vr0dc8meaabaqaciaacaGaaeqabaqabeGadaaakeaacqWGjbqscqGHDisTdaabdiqaaiqbdsgaKzaajaWaaSbaaSqaaiabdohaZbqabaGccqWIpM+zdaqadiqaaiabfs5aeHGaciqb=D8aJzaanaaacaGLOaGaayzkaaGaeS4JPFMafmizaqMbaKaadaWgaaWcbaGaemyAaKgabeaaaOGaay5bSlaawIa7amaaCaaaleqabaGaeGOmaidaaOGaaCzcaiaaxMaacqqGfbqrcqqGXbqCcqqGUaGlcqqGGaaicqqGXaqmcqGGSaalaaa@4A60@

where d^i
 MathType@MTEF@5@5@+=feaafiart1ev1aaatCvAUfKttLearuWrP9MDH5MBPbIqV92AaeXatLxBI9gBaebbnrfifHhDYfgasaacH8akY=wiFfYdH8Gipec8Eeeu0xXdbba9frFj0=OqFfea0dXdd9vqai=hGuQ8kuc9pgc9s8qqaq=dirpe0xb9q8qiLsFr0=vr0=vr0dc8meaabaqaciaacaGaaeqabaqabeGadaaakeaacuWGKbazgaqcamaaBaaaleaacqWGPbqAaeqaaaaa@2F94@, d^s
 MathType@MTEF@5@5@+=feaafiart1ev1aaatCvAUfKttLearuWrP9MDH5MBPbIqV92AaeXatLxBI9gBaebbnrfifHhDYfgasaacH8akY=wiFfYdH8Gipec8Eeeu0xXdbba9frFj0=OqFfea0dXdd9vqai=hGuQ8kuc9pgc9s8qqaq=dirpe0xb9q8qiLsFr0=vr0=vr0dc8meaabaqaciaacaGaaeqabaqabeGadaaakeaacuWGKbazgaqcamaaBaaaleaacqWGZbWCaeqaaaaa@2FA8@ describe the polarization of the incident and scattered light. The Raman tensor Δχ↔
 MathType@MTEF@5@5@+=feaafiart1ev1aaatCvAUfKttLearuWrP9MDH5MBPbIqV92AaeXatLxBI9gBaebbnrfifHhDYfgasaacH8akY=wiFfYdH8Gipec8Eeeu0xXdbba9frFj0=OqFfea0dXdd9vqai=hGuQ8kuc9pgc9s8qqaq=dirpe0xb9q8qiLsFr0=vr0=vr0dc8meaabaqaciaacaGaaeqabaqabeGadaaakeaaiiGacuWFhpWygaqdaaaa@2E7E@ depends on how the polarizability (due to the electromagnetic field of the laser) of the material changes with displacements produced by the vibrational mode.

For the computation of the Raman tensor we generalize the bond polarizability model [21] to a continuum-like model. We assume that the material is composed of bonds between atoms in which each bond has a derivative of the polarizability tensor with components parallel to the bond direction (α∥'
 MathType@MTEF@5@5@+=feaafiart1ev1aaatCvAUfKttLearuWrP9MDH5MBPbIqV92AaeXatLxBI9gBaebbnrfifHhDYfgasaacH8akY=wiFfYdH8Gipec8Eeeu0xXdbba9frFj0=OqFfea0dXdd9vqai=hGuQ8kuc9pgc9s8qqaq=dirpe0xb9q8qiLsFr0=vr0=vr0dc8meaabaqaciaacaGaaeqabaqabeGadaaakeaaiiGacqWFXoqydaqhaaWcbaGaeSyjIafabaGaei4jaCcaaaaa@307D@) and perpendicular (α⊥'
 MathType@MTEF@5@5@+=feaafiart1ev1aaatCvAUfKttLearuWrP9MDH5MBPbIqV92AaeXatLxBI9gBaebbnrfifHhDYfgasaacH8akY=wiFfYdH8Gipec8Eeeu0xXdbba9frFj0=OqFfea0dXdd9vqai=hGuQ8kuc9pgc9s8qqaq=dirpe0xb9q8qiLsFr0=vr0=vr0dc8meaabaqaciaacaGaaeqabaqabeGadaaakeaaiiGacqWFXoqydaqhaaWcbaGaeyyPI4fabaGaei4jaCcaaaaa@3106@) due to changes in bond length. Each bond is assumed to occupy a volume *V*_*b *_and all bonds are assumed to have the same polarizability derivates. The bonds are then averaged over all random orientations. The spirit of the model is to produce a long length-scale theory that averages over many atoms but is derived from a tested atomistic model. The derived change of the polarizability tensor due to the strain field is

Δα↔=αsVb(U↔−13I↔⋅Tr(U↔))+αcVbTr(U↔)I↔.     Eq. 2.
 MathType@MTEF@5@5@+=feaafiart1ev1aaatCvAUfKttLearuWrP9MDH5MBPbIqV92AaeXatLxBI9gBaebbnrfifHhDYfgasaacH8akY=wiFfYdH8Gipec8Eeeu0xXdbba9frFj0=OqFfea0dXdd9vqai=hGuQ8kuc9pgc9s8qqaq=dirpe0xb9q8qiLsFr0=vr0=vr0dc8meaabaqaciaacaGaaeqabaqabeGadaaakeaacqqHuoariiGacuWFXoqygaqdaiabg2da9maalaaabaGae8xSde2aaSbaaSqaaiabdohaZbqabaaakeaacqWGwbGvdaWgaaWcbaGaemOyaigabeaaaaGcdaqadiqaaiqbdwfavzaanaGaeyOeI0YaaSaaaeaacqaIXaqmaeaacqaIZaWmaaGafmysaKKba0aacqGHflY1cqWGubavcqWGYbGCdaqadiqaaiqbdwfavzaanaaacaGLOaGaayzkaaaacaGLOaGaayzkaaGaey4kaSYaaSaaaeaacqWFXoqydaWgaaWcbaGaem4yamgabeaaaOqaaiabdAfawnaaBaaaleaacqWGIbGyaeqaaaaakiabdsfaujabdkhaYnaabmGabaGafmyvauLba0aaaiaawIcacaGLPaaacuWGjbqsgaqdaiabc6caUiaaxMaacaWLjaGaeeyrauKaeeyCaeNaeeOla4IaeeiiaaIaeeOmaiJaeiOla4caaa@5AF8@

The parameters *α*_*s *_and *α*_*c *_are the polarizability changes of the material due to shear strain and compressional strain, respectively. The parameters *α*_*s *_and *α*_*c *_are related to α∥'
 MathType@MTEF@5@5@+=feaafiart1ev1aaatCvAUfKttLearuWrP9MDH5MBPbIqV92AaeXatLxBI9gBaebbnrfifHhDYfgasaacH8akY=wiFfYdH8Gipec8Eeeu0xXdbba9frFj0=OqFfea0dXdd9vqai=hGuQ8kuc9pgc9s8qqaq=dirpe0xb9q8qiLsFr0=vr0=vr0dc8meaabaqaciaacaGaaeqabaqabeGadaaakeaaiiGacqWFXoqydaqhaaWcbaGaeSyjIafabaGaei4jaCcaaaaa@307D@ and α⊥'
 MathType@MTEF@5@5@+=feaafiart1ev1aaatCvAUfKttLearuWrP9MDH5MBPbIqV92AaeXatLxBI9gBaebbnrfifHhDYfgasaacH8akY=wiFfYdH8Gipec8Eeeu0xXdbba9frFj0=OqFfea0dXdd9vqai=hGuQ8kuc9pgc9s8qqaq=dirpe0xb9q8qiLsFr0=vr0=vr0dc8meaabaqaciaacaGaaeqabaqabeGadaaakeaaiiGacqWFXoqydaqhaaWcbaGaeyyPI4fabaGaei4jaCcaaaaa@3106@, but we treat them as parameters. Our calculation only predicts relative (not absolute) intensities, so only the ratio *α*_*c*_/*α*_*s *_is important. We set this ratio to 1/2, which models carbon-carbon bonds well. Only the n = 0 modes depend on this ratio (see Eqs. 3a-c). Details of the model are described in Ref. 22. Integrating Eq. 2 over the volume of the cylindrical viral shell gives the total susceptibly tensor of the viral coat. Since the polarizabilty per unit volume, Eq. 2, is dependant on either cos(*nθ*)or sin(*nθ*), the integral over the angular dependence implies that the total susceptibility tensor in Cartesian coordinates is non zero for only n = 0,1 and 2 modes of the cylindrical shell.

The results are,

I∝(2λ+μ)[13+2αcαs]2Γ02, for n=0;     (Eq. 3A)
 MathType@MTEF@5@5@+=feaafiart1ev1aaatCvAUfKttLearuWrP9MDH5MBPbIqV92AaeXatLxBI9gBaebbnrfifHhDYfgasaacH8akY=wiFfYdH8Gipec8Eeeu0xXdbba9frFj0=OqFfea0dXdd9vqai=hGuQ8kuc9pgc9s8qqaq=dirpe0xb9q8qiLsFr0=vr0=vr0dc8meaabaqaciaacaGaaeqabaqabeGadaaakeaacqWGjbqscqGHDisTdaqadiqaaiabikdaYGGaciab=T7aSjabgUcaRiab=X7aTbGaayjkaiaawMcaamaadmGabaWaaSaaaeaacqaIXaqmaeaacqaIZaWmaaGaey4kaSIaeGOmaiZaaSaaaeaacqWFXoqydaWgaaWcbaGaem4yamgabeaaaOqaaiab=f7aHnaaBaaaleaacqWGZbWCaeqaaaaaaOGaay5waiaaw2faamaaCaaaleqabaGaeGOmaidaaOGaeu4KdC0aa0baaSqaaiabgcdaWaqaaiabgkdaYaaakiabcYcaSiabbccaGiabbAgaMjabb+gaVjabbkhaYjabbccaGiabb6gaUjabg2da9iabicdaWiabcUda7iaaxMaacaWLjaWaaeWaceaacqqGfbqrcqqGXbqCcqqGUaGlcqqGGaaicqqGZaWmcqqGbbqqaiaawIcacaGLPaaaaaa@5AAF@

I∝μ⋅Γ12, for n=1;     (Eq 3B)
 MathType@MTEF@5@5@+=feaafiart1ev1aaatCvAUfKttLearuWrP9MDH5MBPbIqV92AaeXatLxBI9gBaebbnrfifHhDYfgasaacH8akY=wiFfYdH8Gipec8Eeeu0xXdbba9frFj0=OqFfea0dXdd9vqai=hGuQ8kuc9pgc9s8qqaq=dirpe0xb9q8qiLsFr0=vr0=vr0dc8meaabaqaciaacaGaaeqabaqabeGadaaakeaacqWGjbqscqGHDisTiiGacqWF8oqBcqGHflY1cqqHtoWrdaqhaaWcbaGaeGymaedabaGaeGOmaidaaOGaeiilaWIaeeiiaaIaeeOzayMaee4Ba8MaeeOCaiNaeeiiaaIaeeOBa4Maeyypa0JaeGymaeJaei4oaSJaaCzcaiaaxMaadaqadiqaaiabbweafjabbghaXjabbccaGiabbodaZiabbkeacbGaayjkaiaawMcaaaaa@49BF@

I∝(2λ+μ)Γ224, for n=2.     (Eq. 3C)
 MathType@MTEF@5@5@+=feaafiart1ev1aaatCvAUfKttLearuWrP9MDH5MBPbIqV92AaeXatLxBI9gBaebbnrfifHhDYfgasaacH8akY=wiFfYdH8Gipec8Eeeu0xXdbba9frFj0=OqFfea0dXdd9vqai=hGuQ8kuc9pgc9s8qqaq=dirpe0xb9q8qiLsFr0=vr0=vr0dc8meaabaqaciaacaGaaeqabaqabeGadaaakeaacqWGjbqscqGHDisTdaqadiqaaiabikdaYGGaciab=T7aSjabgUcaRiab=X7aTbGaayjkaiaawMcaamaalaaabaGaeu4KdC0aa0baaSqaaiabgkdaYaqaaiabgkdaYaaaaOqaaiabisda0aaacqGGSaalcqqGGaaicqqGMbGzcqqGVbWBcqqGYbGCcqqGGaaicqqGUbGBcqGH9aqpcqaIYaGmcqGGUaGlcaWLjaGaaCzcamaabmGabaGaeeyrauKaeeyCaeNaeeOla4IaeeiiaaIaee4mamJaee4qameacaGLOaGaayzkaaaaaa@4E54@

Here *λ *and *μ *are functions of the scattering angle *θ*, *λ *= ⌊4 - 6 sin^2 ^(*θ*)⌋ *μ *= ⌊14 - sin^2 ^(*θ*)⌋, and Γ_n _are numerically evaluated integrals over the displacement pattern of the modes. The experiments were performed at a scattering angle of 90°. The Γ integrals are over the displacement pattern of the vibration. For example, the n = 0 integral is,

Γ0=∫abω2cl2rdr[AJn(ωr/cl)+BYn(ωr/cl)]
MathType@MTEF@5@5@+=feaafiart1ev1aaatCvAUfKttLearuWrP9MDH5MBPbIqV92AaeXatLxBI9gBaebbnrfifHhDYfgasaacH8akY=wiFfYdH8Gipec8Eeeu0xXdbba9frFj0=OqFfea0dXdd9vqai=hGuQ8kuc9pgc9s8qqaq=dirpe0xb9q8qiLsFr0=vr0=vr0dc8meaabaqaciaacaGaaeqabaqabeGadaaakeaacqqHtoWrdaWgaaWcbaGaeyimaadabeaakiabg2da9maapehabaWaaSaaaeaaiiGacqWFjpWDdaahaaWcbeqaaiabikdaYaaaaOqaaiabdogaJnaaDaaaleaacqWGSbaBaeaacqaIYaGmaaaaaaqaaiabdggaHbqaaiabdkgaIbqdcqGHRiI8aOGaemOCaiNaemizaqMaemOCaiNaei4waSLaemyqaeKaemOsaO0aaSbaaSqaaiabd6gaUbqabaGcdaqadiqaaiab=L8a3jabdkhaYjabc+caViabdogaJjabdYgaSbGaayjkaiaawMcaaiabgUcaRiabdkeacjabdMfaznaaBaaaleaacqWGUbGBaeqaaOWaaeWaceaaiiaacqGFjpWDcqWGYbGCcqGGVaWlcqWGJbWycqWGSbaBaiaawIcacaGLPaaacqGGDbqxaaa@5C00@ where J_n _and Y_n _are Bessel functions.

**Figure 7:**
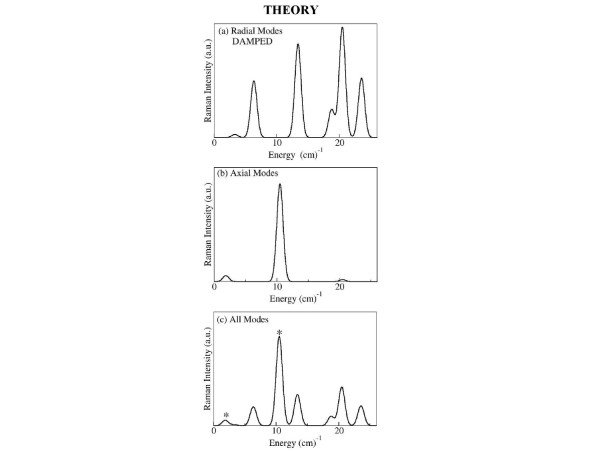
Theoretical Raman Intensity of (a) radial modes; (b) axial modes; (c) all modes. Radial modes are expected to be highly damped by the solution. In (c), the undamped axial modes are indicated by a star (*).

Applying this theoretical foundation to the M13 bacteriophage, we find several peaks in the range of frequency range of 0–25 *cm*^-1^. Figure 7 shows the modes and their Raman intensities (Gaussians broadened by 1 *cm*^-1^). Figure 7a shows the radial models, Figure 7b shows the axial models, and all modes are shown together in Figure 7c. It is important to separate the modes by their character because damping of the two types of modes is expected to be quite different. The radial modes will be highly damped since the atomic displacements of the virus move in or out radially either pushing against the water on the exterior or against the water/DNA solution in the interior cavity of the virus. Axial modes produce displacements of the atoms that are parallel to the axis of the virus and hence should not interact strongly with water. The motion relative to water of the axial modes will be shear motion (rather than compressional as for radial modes) and damping will be marginal. The modes marked with a star (*) in Fig. 7c are axial. We hypothesize that only the axial modes (those with a star) are observable in the Raman experiment because the other (radial) modes are strongly damped.

**Figure 8:**
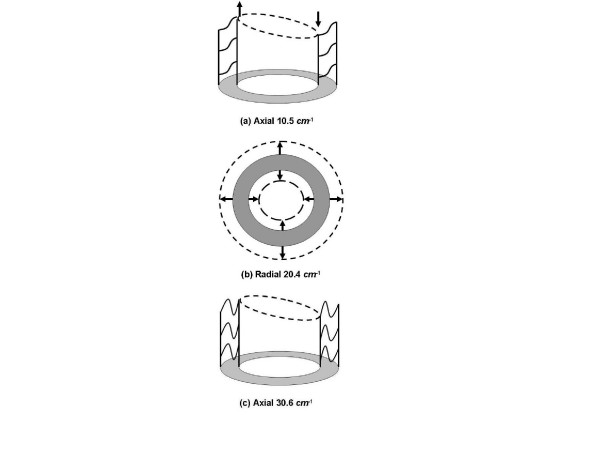
Cross sectional displacement patterns for three low frequency modes with significant Raman intensity. (a) An axial mode at 10.5 *cm*^-1^. This mode we identify as the 8.5 *cm*^-1 ^mode observed in experiment. (b) A radial mode at 20.4 *cm*^-1^, expected to be damped by solution. (c) An axial mode at 30.6 *cm*^-1^.

There are two main Raman intensities predicted in Figure 7c – one at 10.5 *cm*^-1 ^that is axial, and one at 20.4 *cm*^-1 ^that is radial. The mode patterns of these two modes are shown in Figure 8a and 8b. The first main peak at 10.5 *cm*^-1 ^is the largest and corresponds to an n = 1 axial mode of vibration for the cylindrical shell. In this mode each α-helix that comprises the protein coat undergoes axial shearing. Circling about the virus by an angle θ produces a modulation of the displacement by cos(θ) (or sin(θ), as it is doubly degenerate). The mode pattern for one plane of atoms in a plane of the virus is similar to that of a drum-head. The second major peak at 20.4 *cm*^-1 ^corresponds to radial compression/expansion of the virus. The mode has n = 0, so that there is no dependence on the angle θ.

**Figure 9:**
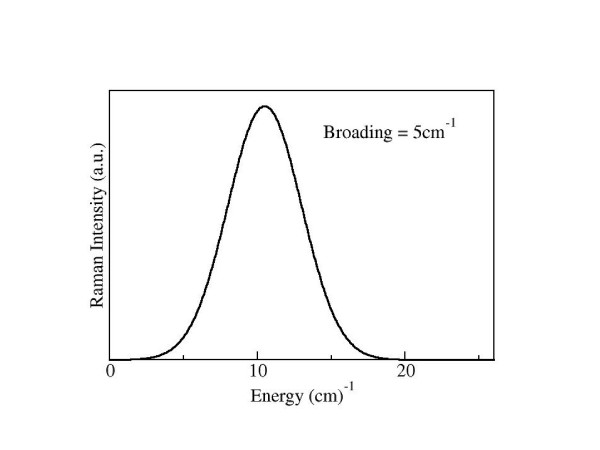
Final theoretical Raman intensity prediction after considering the damping effects of the solvent and the validity of the elastic continuum model.

Our hypothesis for the observed Raman scattering profile is shown in Figure 9. In this figure we have only included the axial modes since they should resonate with little damping. We have included a Gaussian broadening of 5 *cm*^-1^, consistent with inhomogeneous broadening observed in the experiment. The low frequency vibrational mode observed in our Raman measurements (~8.5 *cm*^-1^) agrees remarkably well with the theoretical prediction of a single peak (~10.5 *cm*^-1^) produced by an axial modes.

There are many other modes of higher frequency that are predicted by the continuum model. We do not investigate frequencies higher than 25 *cm*^-1 ^here because either (i) these modes correspond to n>2 and hence are not active in Raman spectroscopy, or (ii) the modes have internal wavelengths of a few Ångstroms which means that they will not be predicted reliably in a continuum model. An example of such a mode is shown in Figure 8c. This mode is just over 30 *cm*^-1 ^and has an internal wavelength of about 10Å. A continuum model averages over many atoms (distances of several bondlengths) and this mode is not expected to be computed accurately. Future work will develop the model further to include atomistic details and coupling of the modes with the solvent. Such extensions will further test the limits of the continuum theory, and probe deeper into our hypothesis concerning the relative importance of damping of the various modes.

In summary, the theory has identified the low frequency modes of vibration of the M13 phage in solution and qualitatively and semi-quantitatively reproduced the observed Raman spectrum. This indicates that Raman spectroscopy is a useful, non-destructive technique for probing the low frequency modes of the virus. These modes are global, being affected by the overall conformational properties of the virus, and will be useful in providing information on how viruses are functionalized for applications in nanotechnology.

We note that Balandin's article [16] calculated the lowest vibrational modes of M13 and TMV viruses in water and air. The authors also pointed out the possible observation of these modes by micro-Raman spectroscopy. They neither (1) performed the micro-Raman experiments; nor (2) calculated/predicted the Raman intensity in a quantitative way. On the other hand, our present work is the *first *experimental results reported in the literature so far for the low vibrational modes of M13 phages. In addition, we have used bond-polarizability model to calculate/predict the relative strength of various Raman active modes in M13 phages. By using this theoretical model, we were able to not only explain the frequency of the mode observed in our Raman experiments but also identify it to be the axial torsion mode of the protein coat of M13 phages. Therefore, our current contributions significantly advance the work of Balandin's.

## Conclusion

Low frequency vibrational modes of the M13 phage have been studied by Raman spectroscopy. The observed vibrational mode at 8.5 *cm*^-1 ^agrees well with theoretical model calculations that are based on an elastic continuum model and a bond polarizability model with Raman selection rules. With the help of theoretical data, we have determined that the observed Raman mode corresponds to an axial (drum-head-like) vibrational mode of the M13 phage protein coat which suffers minimal damping in the exterior solvent. The differences in Raman spectra can be used to monitor the coating of virus with some other materials and nano-assembly process, such as attaching a carbon nanotube or quantum dots. Our results suggest that Raman spectroscopy is a feasible, non-destructive technique for probing the process of virus functionalization. Bacteriophage M13 is one of the simplest paradigms for viral capsids, and the characterization of its low frequency vibrational modes is a significant step towards the use of these modes for functionalization studies.

## References

[B1] Liu JL, Wu WG, Balandin A, Jin G, Wang KL (1999). Intersubband Absorption in boron-doped multiple Ge quantum dot. Appl Phys Lett.

[B2] Liu JL, Wu WG, Balandin A, Lin G, Luo YH, Thomas SG, Lu Y, Wang KL (1999). Observation of inter-sub-level transitions in modulation-doped Ge quantum dots. Appl Phys Lett.

[B3] Kamins TI, Williams RS (1997). Lithographic positioning of self-assembled Ge islands on Si (001). Appl Phys Lett.

[B4] Balandin A, Jin G, Wang KL (2000). Issues of practical realization of a quantum dot register for quantum computing. J Electronics Materials.

[B5] Bandyopadhyay S, Miller AE, Chang HC, Banerjee G, Yue DE, Ricker RE, Jones S, Eastman JA, Chandrasekhar M (1996). Electrochemically assembled quasi-periodic quantum dot arrays. Nanotechnology.

[B6] Balandin A, Wang KL, Kouklin N, Bandyopadhyay S (2000). Raman spectroscopy of electrochemically self-assembled CdS quantum dots. Appl Phys Lett.

[B7] Shenton W, Douglas, Young TM, Stubbs G, Mann S (1999). Inorganic-organic nanotube composites from template mineralzation of tobacco mosaic virus. Adv Mater.

[B8] Flynn CE, Lee SW, Peelle BR, Belcher AM (2003). Viruses asvehicles for growth, organization and assembly of materials. Acta Materialia.

[B9] Mao C, Solis DJ, Reiss BD, Kottmann SD, Sweeney RY, Hayhurst A, Georgiou G, Iverson B, Belcher AM (2004). Virus-based toolkit for the directed synthesis of magnetic and semiconductoring nanowires. Science.

[B10] Knez M, Bittner AM, Boes F, Wege C, Jeske H, Maiss E, Kern K (2003). Biotemplate of 3-nm nickel and cobalt nanowires. Nano letters.

[B11] Knez M, Sumser M, Bittner AM, Wege C, Jeske H, Martin TP, Kern K (2004). Spatially selective nucleation of metal clusters on the tobacco mosaic viruses. Adv Funct Mater.

[B12] Tuma R, Thomas GJ, Chalmers JM, Griffiths PR (2002). Raman Spectroscopy of Viruses, in Handbook of Vibrational Spectroscopoy.

[B13] Eisenstark A, Maramorosch K, Koprowski H (1967). Bacteriophage techniques. Methods in virology.

[B14] Tsen KT, Wald KR, Ruf Tobias, Yu PY, Morkoc H (1991). Electron-optical phonon interactions in ultrathin GaAs-AlAs multiple quantum well structures. Phys Rev Lett.

[B15] Marvin DA, Welsh LC, Symmons MF, Scott WRP, Strauss SK (2006). Molecular structure and fd (f1, M13) filamentous bacteriophage refined with respect to X-ray fibre diffraction and solid-state NMR data supports specific models of phage assembly at the bacterial membrane. J Mol Biol.

[B16] Balandin A, Fonoberov VA (2005). Vibrational modes ofNano-Template Viruses. Journal of Biomedical Nanotechnology.

[B17] Graff KF (1991). Wave motion in elastic solids.

[B18] Landau LD, Lifshitz EM (1986). Theory of Elasticity.

[B19] Tachibana M, Kojima K, Ikuyama R, Kobayashi Y, Ataka M (2000). Sound velocity and dynamic elastic constants of lysozyme single crystals. Chem Phys Lett.

[B20] Yu PY, Cardona M (1999). Fundamentals of Semiconductors – Physics and Materials Properties.

[B21] Go S, Bilz H, Cardona M (1975). Bond Charge, BondPolarizability, and Phonon Spectra in Semiconductors. Phys Rev Lett.

[B22] Dykeman EC, Tsen KT, Sankey OF

